# Colonization patterns of *Enterococcus cecorum* in two different broiler production cycles detected with a newly developed quantitative real-time PCR

**DOI:** 10.1186/s12866-017-1021-7

**Published:** 2017-05-05

**Authors:** Arne Jung, Henning Petersen, Lydia Teske, Silke Rautenschlein

**Affiliations:** Clinic for Poultry, University of Veterinary Medicine Hannover, Buenteweg 17, D-30559 Hannover, Germany

**Keywords:** Enterococcus cecorum infection, Quantitative real-time PCR, Colonization, Broiler chicken

## Abstract

**Background:**

Although *Enterococcus cecorum* (EC) infection is one of the most important bacterial diseases in modern broiler chickens today, many aspects of epidemiology and pathogenesis are still unknown. There is a need for better detection methods for EC than classical cultivation. In the present study, we describe the validation and application of a newly developed quantitative TaqMan real-time PCR (qPCR) assay based on the 16S–rRNA-gene for the detection of EC.

**Results:**

Fifty EC strains isolated from 12 different animal species were detected with the assay, while none of the other 26 examined bacterial species were tested positive during validation procedure. The detection limit of the PCR was 6.25 CFU/ml PBS. The qPCR assay was also considerably more sensitive using intestine and organ samples than the classical cultivation method. Field application of the PCR setup was tested comparing two different broiler production cycles on one farm: in cycle I broilers showed signs of enterococcal spondylitis (ES) from day 24 post hatch onwards while broilers in cycle II developed no ES. Two totally different colonization patterns were found in the two cycles with the qPCR using cloacal swabs. Animals in cycle I showed significantly (*P* ≤ 0.05) higher detection rates of EC at the day of placement and throughout the cycle than broilers of cycle II. Additionally, significantly higher detection rates were found in the cecum compared to duodenum, jejunum and ileum within one cycle.

**Conclusions:**

The new qPCR for EC is highly specific, more sensitive than classical cultivation and was able to show differences in colonization in a broiler cycle with later EC disease outbreak compared to a healthy cycle. These findings may be explained by infection with different strains, pathogenic EC isolates are probably more effective in colonization than commensal isolates. A high correlation was found between qPCR results from cecum and cloacal swabs in this study, indicating that cloacal swabs can be used to examine intestinal colonization of broilers with EC. The new qPCR significantly improves the diagnostic of EC infections and may help to answer open questions concerning epidemiology and pathogenesis.

## Background

*Enterococcus cecorum* (EC) is the main causative agent of enterococcal spondylitis (ES) in broilers and can be designated as an emerging avian pathogen. In the last 10 years it became one of the most important bacterial diseases in commercial broiler operations and outbreaks were reported from many different countries worldwide [1–10]. Affected flocks exhibit hind limb paresis in the last third of the growing period. During necropsy, birds show pericarditis and hepatitis followed by spondylitis of the free (6th) thoracic vertebra and femoral head necrosis as the most prominent pathological changes. Cumulative mortality reaches 5–15% in one cycle [2, 6, 11] and can be accompanied with higher condemnation rates at the processing plant [2]. Additionally, EC can infect Peking ducks [12, 13].

In EC infected broiler flocks, generalized infections with pericarditis and hepatitis were detected as early as 6 days post hatch, while spondylitis and femoral head necrosis was found at the end of the cycle [2]. Therefore, septicemia seems to be one step in the pathogenesis of ES. It was speculated that during the fast growth of modern broiler chickens, micro-lesions may develop at the weight-bearing free thoracic vertebra and femoral heads and EC infect these lesions during bacteremia [7, 14, 15]. Recently, it was shown that only broilers with advanced osteochondrosis dissecans in the free thoracic vertebra developed spondylitis after oral infection with a virulent EC strain [16]. Furthermore, in broilers which developed ES, EC was isolated from the intestine in the first week of life, while in non-affected flocks it was not detected before the third week [16]. These results show that intestinal colonization is an important part of the pathogenesis of ES and precedes septicemia. In the mentioned study, the whole intestinal tract was homogenized, so there is no information which part of the intestine was colonized by EC. In another study, EC was isolated from 57% of the crops, 18% of the small intestines and 3% of the ceca in 3–4 week old healthy broilers as well as from 43% of the crops, 64% of the small intestines and 68% of the ceca in over 12 week old healthy broiler and layer parents [17]. Based on these data, EC seems to colonize the small intestine first, before the cecum is infected. However, it should be considered that this study was done in 1991, before EC associated disease was recognized as a problem. Virulent EC strains may show different colonization patterns.

The source of EC infection was never detected for broiler flocks, neither direct vertical transmission could be demonstrated nor could EC be detected in the hatchery [6, 18]. The yolk sac from embryos with different ages, eggshells, chick dander samples, water samples, chick paper, different surface samples including incubator airflow and hatcher airflow as well as air samples from the hatchery were negative for EC. Nevertheless, EC was repeatedly isolated from broiler parent flocks, which can also develop disease [6, 15, 18]. Furthermore, ES was diagnosed in chickens from the first growing cycle in newly built broiler houses (personal communication with other poultry veterinarians). Therefore, the hatchery can still not be excluded as the source of infection. Sometimes ES outbreaks are repeatedly observed in successive broiler cycles, indicating an on-farm reservoir. Despite these observations, samples from broiler houses such as feed and water samples, litter, air vents and mice from rodent traps were tested negative for EC [6, 16]. However, in all mentioned studies which were done in the hatcheries as well as in broiler houses, classical culturing methods were used for the detection of EC. The goal of this study was the development of a quantitative real-time polymerase chain reaction (qPCR) for the detection of EC. For validation of the PCR, the colonization pattern of EC in two broiler cycles of one farm was monitored from placement to slaughter.

## Methods

### Bacterial strains

Bacterial field strains included in this study are listed in Table 1. EC strains were isolated from 1995 to 2015 [19]. The other bacterial strains were isolated from 1990 to 2016. Bacterial reference strains included in this study are listed in Table 2. All strains were archived as pure cultures using the cryobank system (Mast Diagnostica GmbH, Reinfeld, Germany). DNA isolation was done using the boiling method. Briefly, pure subcultures of each strain were produced and one loop culture material was mixed into 500 μl molecular biology grade water (Sigma-Aldrich, München, Germany. Samples were incubated at 95 °C and 500 rpm in a heating block with shaking function and centrifuged at 13.000 x g for 5 min. The supernatant was archived at −20 °C. All field isolates were reliably identified by partial sequencing of the 16S–rRNA-gene [20–22] at Microsynth AG (Lindau, Germany).

**Table 1:** Bacterial field strains included in this study

Bacterial species	Animal species	Number of isolates
*Enterococcus cecorum*	Broiler	15
	Pekin duck	11
	Laying hen	6
	Turkey	5
	Pigeon	4
	Cattle	2
	Swine	2
	Budgerigar	1
	Goose	1
	Human	1
	Muscovy duck	1
	Swan	1
*Enterococcus columbae*	Pigeon	4
*Enterococcus faecalis*	Broiler	2
	Goose	1
	Pekin duck	1
*Enterococcus faecium*	Broiler	2
	Turkey	2
	Laying hen	1
	Pekin duck	1
*Enterococcus gallinarum*	African grey parrot	1
	Broiler	1
	Goose	1
*Enterococcus hirae*	Broiler	1
	Macaw	1
*Lactobacillus salivarius*	Budgerigar	1
	Turkey	1
*Lactococcus garvieae*	Pekin duck	1
*Lactococcus lactis*	Goose	1
	Pekin duck	1
*Streptococcus alactolyticus*	Pekin duck	2
*Streptococcus anginosus*	Laying hen	1
*Streptococcus gallolyticus*	Broiler	1
	Pekin duck	1
	Turkey	1
*Streptococcus pluranimalium*	Pekin duck	1

**Table 2:** Bacterial reference strains included in this study

Bacterial species	Strain collection numbers
*Acinetobacter johnsonii*	DSM 6963
*Aeromonas hydrophila*	DSM 30187, ATCC 7966
*Citrobacter freundii*	DSM 30039
*Enterococcus avium*	DSM 20063
*Enterococcus casseliflavus*	DSM 20680, CCM 2478
*Enterococcus cecorum*	DSM 20682, ATCC 43198, NCDO 2674
*Enterococcus durans*	LMG 12903, ATCC 11576
*Enterococcus faecium*	DSM 2146, CCM 2308
*Enterococcus hirae*	DSM 20160
*Escherichia coli*	DSM 30083, ATCC 11775
*Pseudomonas putida*	DSM 291
*Staphylococcus aureus*	DSM 1104, ATCC 25923
*Staphylococcus epidermidis*	DSM 20044, ATCC 14990
*Staphylococcus kloosii*	DSM 20676, ATCC 43959
*Stapylococcus intermedius*	DSM 20373, ATCC 29663
*Streptococcus equi ssp.equi*	DSM 20561, ATCC 33398
*Yersinia pseudotuberculosis*	DSM 8992

### Quantitative real-time PCR (qPCR)

Primers and TaqMan probe were designed based on the 16S–rRNA-gene. Allele sequences from *Enterococcus* (*E.*) *avium, E. casseliflavus, E. cecorum, E. columbae, E. durans, E. faecalis, E. faecium, E. gallinarum and E. hirae* were downloaded from GenBank® [23] and subsequently aligned using ClustalW multiple sequence alignment tool (MEGA7 v7.0.14). EC-specific primers (qEcec_for: 5′-ACAGGTGCTAATACCGCATAAT-3′; qEcec_rev: 5′-CCCACCAACTAGCTAATGCAC-3′ and probe (qEcec_probe: 5′-FAM-ACCGCATGGTAGATGGATGAAAGGC-BHQ1–3′) were selected for validation. For the optimized qPCR, the PerfeCTa qPCR ToughMix (Quanta Biosciences, Beverly, USA) was used. Each qPCR mixture included 0.5 μl EGFP-1-F, 0.5 μl EGFP-10-R, 0.5 μl EGFP-HEX (primers and probe for internal control), 1.0 μl of each primer and 0.5 μl of the probe (all in a concentration of 10 pmmol/μl), 10 μl PerfeCTa qPCR ToughMix, 0.5 μl internal control Intype IC-DNS (Qiagen; Hilden, Germany) and 2 μl template in a total volume of 20 μl. PCR amplification was carried out on a MxPro3005P cycler (Stratagene; Agilent Technologies, Waldronn, Germany) with following cycling conditions: initial denaturation at 95 °C for 10 min followed by 45 cycles of 95 °C for 15 s and 60 °C for 60 s. Ct values above 40 were considered negative. Data are presented as 40-Ct.

### Specificity and analytical sensitivity of the qPCR

Eighty-four bacterial field strains and 17 bacterial reference strains listed in Tables 1 and 2 were used to determine the specificity of the qPCR assay. For assessment of the lower limit of detection (LLD), one colony of an 18 h incubated EC strain was mixed into 100 ml phosphate buffered saline (PBS) and a 10-fold dilution series was prepared using PBS. The 1000-fold diluted suspension was used to prepare a 2-fold serial dilution. Two μl template of the 1000-fold up to the 16,000-fold dilution was then used in the qPCR to determine the LLD of the assay and 100 μl of the suspensions were inoculated onto Columbia sheep blood (CSB) agar plates in parallel to detect the bacterial concentration in CFU per ml.

### Bacterial cultivation

Selected swab samples were inoculated onto Colistin Nalidixic Acid (CNA) agar (Oxoid GmbH, Wesel, Germany) and incubated for 24 h at 37 °C in a CO_2_-enriched atmosphere (5% CO_2_). After 24 h, agar plates were monitored for bacterial growth, results were documented and pure subcultures from single colonies were produced on CNA agar. Isolates were identified as EC using the new qPCR.

### Collection of field samples

Two broiler cycles on the same farm were sampled in 2016. Cycle I started in July 2016, cycle II in November 2016 respectively. In each cycle, 16,400 broilers (Ross 308) were equally divided into two groups of 8200 birds and placed in two compartments of a broiler house, separated by a lightweight wall but connected by a door for personnel. Maximum stocking rate at the last day before slaughter was 35 kg/m^2^ according to requirements of the German “Initiative Tierwohl” label for broilers. Broiler chickens were vaccinated in the hatchery with Poulvac® IB Primer (Zoetis Deutschland GmbH, Berlin, Germany), administered as coarse spray, and were obtained from BWE hatchery (Visbek, Germany), placed on wood shavings and fed with 4-phase pelleted feed. All broilers in cycle I/cycle II received coccidiostats as follows: the starter feed contained Decoquinat (20 mg/kg feed)/Narasin (60 mg/kg feed) and Nicarbazin (125 mg/kg feed), grower feed 1 (slow growth) was used from day 8/day 6 post hatch (ph), grower feed 2 (slow growth) was used from day 23/day 21 ph and both contained Monensin-Sodium (100 mg/kg feed)/Narasin (60 mg/kg feed). Finisher feed was used from day 30/day 27 ph and was free from anticoccidials. All broilers in cycle I/cycle II were vaccinated as follows: day 12/day 12 ph Poulvac® ND Hitchner B1 (Zoetis), day 17/day 18 ph AviPro® Precise (Elanco, Cuxhaven, Germany) and day 19/day 20 ph Nobilis® Ma5 (MSD Tiergesundheit, Unterschleißheim, Germany).

In cycle I, lameness was recognized at day 24 ph in the flock and EC was isolated from one broiler with pericarditis and hepatitis. At day 27 ph, spondylitis and femoral head necrosis was found in 13 out of 24 submitted animals and EC was isolated from these broilers in pure culture. Therefore, all broilers were medicated from day 27 to day 31 ph against EC infection with Suramox 1000 mg/g (Virbac Tierarzneimittel GmbH, Bad Oldesloe, Germany) via the drinking water. No clinical signs were detected in the broilers throughout cycle II. Broilers were slaughtered at day 34 ph in both cycles. The overall mortality in growing cycles I and II was 3.01%/3.58%, rejection rate at the processing plant was 1.47%/2.32% and the mean weight of processable broilers was 2063 g/2085 g respectively.

Animals were sampled with sterile cotton swabs which were stored at −80 °C. At the day of arrival of the chickens on the farm (day 0), cloacal swabs were taken from 20 broilers before placement of the chicks in each cycle. In cycle I, dead and moribund animals were submitted for necropsy at days 3, 6, 10, 13, 17, 20, 24, 27 and 31 ph. Following numbers of animals were sampled with cloacal swabs at necropsy days in cycle I respectively: 10, 8, 4, 9, 10, 9, 7, 10 and 8. Additionally, swaps from duodenum, jejunum, ileum and cecum were collected and swabs from femoral heads and free thoracic vertebrae were collected at day 31 ph. In cycle II, dead and moribund animals were submitted for necropsy at days 4, 7, 11, 14, 18, 21, 25, 28 and 32 ph. Following numbers of animals were sampled with cloacal swabs at necropsy days in cycle II respectively: 10, 10, 10, 10, 4, 9, 10, 7 and 10. Additionally, swaps from duodenum, jejunum, ileum and caecum were collected.

DNA was isolated from the swabs using the InnuPrep DNA Mini-Kit (Analytik Jena AG, Jena, Germany) following manufacturer’s instructions.

### Statistics

For comparison of Ct values from cloacal swabs of both cycles, Wilcoxon Rank-Sum Test was used. Comparison of Ct values from different parts of the intestine in each cycle was done using Kruskal-Wallis Test with Dunn’s all-pairwise comparisons as post hoc-test. Correlation of Ct values from cecum and cloacal swabs was calculated using Spearman’s rank correlation. For calculation of correlation, data from days 3, 13,17, 24 and 31 ph of cycle I and data from days 4, 7, 14, 18 and 32 of cycle II were included, in total 81 cases. All calculations were done using Statistix 10 (Analytical Software, Tallahassee, Florida, USA). Differences in all statistical tests were considered significant at *P* ≤ 0.05.

## Results

### Specificity and analytical sensitivity of qPCR

In the specificity test, all 50 EC strains were positive, while none of the other 26 examined bacterial species generated a positive result using the new qPCR assay. The lower limit of detection (LLD) of the assay was 6.25 CFU/ml. The introduction of the internal control had no negative effect on the LLD of the qPCR (data not shown).

### Examination of field samples via qPCR

**Fig. 1:**
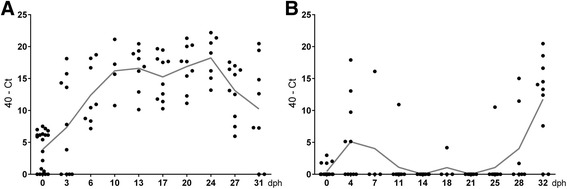
40-Ct values of cloacal swabs of broilers sampled at different days post hatch (dph) detected with the newly developed quantitative PCR for *Enterococcus cecorum* (EC). *n* = 4 to 20; data are presented as scatter plots with connected means. **a** Cycle I, which developed EC-associated disease. **b** Cycle II, which developed no EC-associated disease

In total, 540 swab samples were examined via qPCR for EC. EC colonization was compared between cycle I and cycle II using 194 cloacal swabs. Totally different EC colonization patterns were recognized in both cycles (Fig. 1a and b). In cycle I, EC was already detected in relatively high amounts via qPCR at the day of arrival of the chickens on the farm (day 0). From day 6 onwards, all animals were positive until the end of the cycle. In cycle II, broilers were mostly negative until the last week, when they were colonized with EC. At days 0/0, 10/11, 13/14, 17/18, 17/18, 20/21, 24/25 and 27/28, EC detection rates were significantly higher (*P* ≤ 0.05; Wilcoxon rank-sum test) in broilers of cycle I compared with cycle II, respectively.

**Fig. 2:**
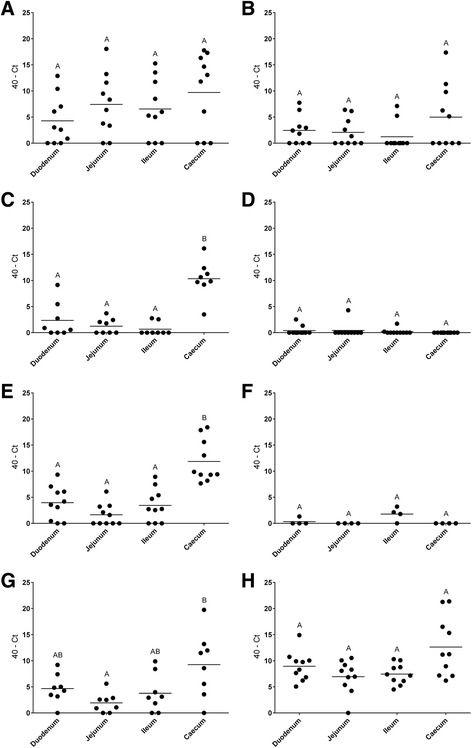
40-Ct values of swabs from duodenum, jejunum, ileum and cecum of broilers sampled at different days post hatch (dph) detected with the newly developed quantitative PCR for *Enterococcus cecorum* (EC). *n* = 4 to 10. **a**, **c**, **e** and **g**. Days 3, 13, 17 and 31 ph of cycle I, which developed EC-associated disease. **b**, **d**, **f** and **h**. Days 4, 14, 18 and 32 ph of cycle II, which developed no EC-associated disease. Different uppercase superscript letters indicate significant differences between groups (*P* ≤ 0.05; Kruskal-Wallis Test, post hoc-test Dunn’s All-Pairwise Comparisons)

We have also analyzed EC colonization of different parts of the intestine in both broiler production cycles (Fig. 2a to h). In broilers of cycle I, significantly higher amounts of EC were detected in the cecum compared to duodenum, jejunum and ileum at days 13, 17 and 31 ph via qPCR (*P* ≤ 0.05; Kruskal-Wallis Test, post hoc-test Dunn’s All-Pairwise Comparisons). In cycle II, no significant differences were found concerning EC detection between different parts of the intestine. In broilers of cycle II, detection rates of EC in all parts of the intestine at days 4, 14 and 18 were lower than in cycle I. Only at day 32, EC detection was comparable to cycle I.

For qPCR results from cecum and cloacal swabs, a Spearman correlation of +0.77 was calculated, which means that qPCR results from cecum and cloacal swabs are highly positively correlated.

### Comparison of qPCR and bacterial cultivation

EC detection via qPCR and conventional cultivation technique in field samples were compared using 8 swab samples from femoral heads and free thoracic vertebrae from day 31 ph in cycle I and 10 cecum samples from day 32 ph in cycle II. In 4 vertebral samples, massive growth of *Enterococcus*-like bacteria were found on the CNA agar plates, while no growth was detected in all other femoral head and vertebral samples. In 6 cecal samples, *Enterococcus*-like bacteria were found on the CNA agar plates, while in the other cecal samples only non-*Enterococcus* colony types were detected. All *Enterococcus*-like cultures looked like pure cultures, therefore subcultures were produced from single colonies. All 10 subcultures were examined using the EC specific qPCR. All 4 vertebral samples and two cecal samples were identified as EC (Table 3). The other cecal strains were negative via qPCR and subsequently identified as *Enterococcus faecalis* (2×), *Enterococcus hirae* and *Enterococcus gallinarum* using partial sequencing of the 16S–rRNA gene [21]. In only 6 out of 26 samples EC was isolated with the cultivation method, while only 2 of the samples were negative with qPCR (Table 3). All samples which were positive by cultivation had 40-Ct values above 15.

**Table 3:** Comparison of qPCR and conventional isolation of *Enterococcus cecorum*

Cycle	dph^a^	broiler ID	Sample	40-Ct	Conventional isolation
I	31	1	Femoral head	0.3	-
I	31	1	Free thoracic vertebra	17.0	+
I	31	2	Femoral head	1.0	-
I	31	2	Free thoracic vertebra	7.7	-
I	31	3	Femoral head	0.3	-
I	31	3	Free thoracic vertebra	0.0	-
I	31	4	Femoral head	2.5	-
I	31	4	Free thoracic vertebra	2.6	-
I	31	5	Femoral head	5.4	-
I	31	5	Free thoracic vertebra	20.3	+
I	31	6	Femoral head	4.8	-
I	31	6	Free thoracic vertebra	8.2	-
I	31	7	Femoral head	2.8	-
I	31	7	Free thoracic vertebra	17.4	+
I	31	8	Femoral head	0.0	-
I	31	8	Free thoracic vertebra	17.4	+
II	32	1	Cecum	21.3	+
II	32	2	Cecum	21.4	-
II	32	3	Cecum	11.2	-
II	32	4	Cecum	16.5	+
II	32	5	Cecum	15.3	-
II	32	6	Cecum	7.1	-
II	32	7	Cecum	8.9	-
II	32	8	Cecum	7.2	-
II	32	9	Cecum	6.2	-
II	32	10	Cecum	11.2	-

^a^Days post hatch

## Discussion

The newly developed qPCR TaqMan assay for the detection of EC is highly specific, as shown during our validation procedure with many different bacterial species. All tested EC strains, pathogenic and also commensal isolates from different animal species were positive using the qPCR assay. Closely related bacteria, such as other species from the genus *Enterococcus*, but also genetically distant species were tested negative with the assay. The analytical sensitivity determined using colony material mixed in PBS probably underestimates the real analytical sensitivity of the qPCR, because common DNA isolation procedures are not efficient in recovering DNA in low concentrations [24]. However, the analytical sensitivity of the qPCR assay was much higher than conventional isolation of EC from femoral heads, vertebrae and intestine. This correlates with experiences from examination of field samples from poultry, where the isolation of enterococci from chronic cases sometimes fails (own observations and personal communication with other laboratories). One additional reason for negative isolation results from highly EC positive cecum samples probably was the similar colony morphology of different *Enterococcus* species and the production of subcultures from single colonies only. Additionally, PCR assays in general detect parts of the genome of microorganisms, whereas cultivation methods only detect living microorganisms which are able to proliferate and are therefore less sensitive. One conventional PCR setup based on the superoxide dismutase (sodA) gene was already published for EC [25]. However, the qPCR described in this study allows real-time quantification of EC and therefore a much broader application. Many aspects of EC epidemiology and pathogenesis are still unknown and this assay may help to answer some of the remaining questions, for example whether broilers bring EC from the hatcheries or are infected from an on-farm reservoir.

In general, marked differences concerning EC colonization were found between broiler production cycle I (EC-associated disease) and cycle II (no EC-associated disease). Already at the day of arrival (day 0), broiler chicks in cycle I showed significant higher detection rates of EC than broilers in cycle II and remained positive until the end of the cycle. Broilers in cycle II were not colonized before week 3 of the cycle (Fig. 1a and b). This was also shown in another study which used selective enrichment broth containing different antibiotics [16]. However, this method may be problematic for detection of strains without resistance against the used antibiotic substances. The reasons for the divergent colonization patterns in the two different production cycles may be explained by infection with different EC strains. Intestinal EC strains from broiler flocks without ES formed a single cluster using PFGE, while 45% of intestinal strains from broiler flocks with ES were located in the cluster with spleen and vertebral isolates [16]. Using whole genome sequencing, an enterococcal polysaccharide antigen (epa)-like locus was found in pathogenic but not in commensal EC strains [26]. This locus is important for translocation from the intestinal tract [27]. In the first week of cycle II, EC was demonstrated in cloacal swabs in some animals, but was not able to maintain colonization. It may be hypothesized that commensal strains lack certain surface proteins which are important for colonization of the intestine of very young broilers.

In this study, detection of EC in different parts of the intestine was compared. Highest concentration of EC was found in the cecum, but it was also detected in duodenum, jejunum and ileum (Fig. 2a to h). Interestingly, at the beginning of colonization in cycle I and II, EC was detected with higher rates in the smaller intestine (Fig. 2a and h). This probably shows the oral uptake and passage through different parts of the intestine until the cecum is colonized, independent of the virulence of the strain.

An important finding was significant higher detection rates of EC in broilers of the diseased cycle I compared to the healthy cycle II already before placement of the chicks in the house. To our best knowledge this is the first detection of EC in newly hatched chicks. This probably means that transmission from the hatchery to the broiler house is more likely than an on-farm reservoir of EC. However, these results have to be verified by examination of more field samples. Additionally, the described qPCR setup may be used as a screening tool for examination of chicks at placement in the broiler house. Higher EC detection rates may indicate later EC infection in the respective broiler cycle. Cloacal swabs of living chicks are sufficient for examination of EC, because we showed in this study that qPCR results of ceca and cloacal swabs are highly correlated which each other. Other possible applications for the qPCR are for example surveillance of broiler parent flocks or hatcheries.

As EC is one of the most important bacterial pathogens in broiler production today, there is a need for better diagnostic tools. In this study, we describe the validation and application of a quantitative real-time PCR for the detection of EC. We showed that colonization patterns of EC in a broiler flock with an EC disease outbreak is totally different from a healthy cycle. The new qPCR assay may be used for studies on epidemiology and pathogenesis of EC.

## Abbreviations

DNA: Deoxyribonucleic acid
EC: *Enterococcus cecorum*
ES: Enterococcal spondylitis
LLD: Lower limit of detection
ph: Post hatch
qPCR: Quantitative polymerase chain reaction
rRNA: Ribosomal ribonucleic acid
